# Pharmacogenomic considerations for repurposing of dexamethasone as a potential drug against SARS-CoV-2 infection

**DOI:** 10.2217/pme-2020-0183

**Published:** 2021-06-04

**Authors:** Manik Vohra, Anu Radha Sharma, Kapaettu Satyamoorthy, Padmalatha S Rai

**Affiliations:** 1 ^1^Department of Biotechnology, Manipal School of Life Sciences, Manipal Academy of Higher Education, Manipal 576104, Karnataka, India; 2 ^2^Department of Cell & Molecular Biology, Manipal School of Life Sciences, Manipal Academy of Higher Education, Manipal 576104, Karnataka, India

**Keywords:** COVID-19, dexamethasone, metabolome, pharmacogenetics, SARS-CoV-2, transcriptome

## Abstract

Immunomodulatory and analgesic effects of dexamethasone are clinically well established, and this synthetic corticosteroid acts as an agonist of glucocorticoid receptors. Early results of the RECOVERY Trial from the United Kingdom and others suggest certain benefits of dexamethasone against COVID-19 chronic patients. The efforts have been acknowledged by World Health Organization with an interim guideline to use in patients with a severe and critical illness. The inherent genetic variations in genes such as *CYP3A5*, *NR3C1*, *NR3C2*, etc., involved in the pharmacokinetic and pharmacodynamic processes may influence dexamethasone’s effects as an anti-inflammatory drug. Besides, the drug may influence transcriptome or metabolic changes in the individuals. In the present review, we summarize the reported genetic variations that impact dexamethasone response and discuss dexamethasone-induced changes in transcriptome and metabolome that may influence potential treatment outcome against COVID-19.

## Lay abstract

The surge of COVID-19 cases has increased the need for the development of a cure. This has pushed the barriers of the regulatory controls for randomized controlled trials. There has been the usage of immunomodulatory drugs, such as dexamethasone, with promising results in severe COVID-19 patients to reduce mortality. However, there is a need to consider the inherent genetic factors of an individual that may influence the dexamethasone drug’s metabolism and action. To understand this, there is a need to evaluate the genes involved in the pharmacokinetics and pharmacodynamic pathways of the drug and study the effects of the drug. This will aid in choosing the right individuals who will benefit from the therapy. Hence, the present review summarized the reported genetic variations that impact dexamethasone drug response.

## Background

The threat due to the outbreak of coronavirus disease 2019 (COVID-19) caused by the new coronavirus (SARS-CoV-2) infection is a critical global concern. The present understanding of SARS-CoV-2 infection suggests three different phases, which include an asymptomatic incubation period (Phase I), a nonsevere symptomatic period with the presence of virus (Phase II) and high viral load causing severe respiratory symptoms (Phase III) [1]. On 16 June 2020, World Health Organization (WHO) acknowledged the use of anti-inflammatory drug dexamethasone on critical COVID-19 patients based on the results from the RECOVERY trial conducted in the United Kingdom [2]. The preliminary results suggest that individuals with severe and critical illness may benefit from the drug, unlike the patients with mild symptoms [2]. Dexamethasone in SARS-CoV-2 affected individuals is prompted by the severe inflammation due to several cytokines produced in large excess.

Dexamethasone has been in use since the 1960s and was listed in the WHO Model List of Essential Medicines in 1977 [3–5]. The steroid drug dexamethasone is made synthetically and, like other corticoids, affects the immune system. Dexamethasone has been widely used in conditions with inflammation. These include systemic lupus, rheumatoid arthritis, acute gouty arthritis, ulcerative colitis, psoriatic arthritis and Crohn’s disease [3,6]. Dexamethasone is also used in allergic conditions such as allergic rhinitis, bronchial asthma, contact and atopic dermatitis and drug-induced dermatitis when patients fail to respond other treatments [7,8]. Dexamethasone is also used to treat skin conditions such as pemphigus, dermatitis herpetiformis, severe seborrheic dermatitis and severe psoriasis [9,10].

Despite the fact that preliminary results of SARS-CoV-2 infection treatment with dexamethasone are encouraging, there is a need for caution as the drug trial is still in preliminary stages. Khan *et al.* (2020) suggested through computational modeling that the main protease (M^pro^, also known as 3CL^pro^) of SARS-CoV-2 protein binds tightly to the dexamethasone [11]. M^pro^ cleaves the viral polyproteins, generating 12 nonstructural proteins (Nsp4-Nsp16), including the RNA-dependent RNA polymerase (RdRp, Nsp12) and the helicase (Nsp13). Inhibition of M^pro^ would prevent the virus from replication and, therefore, constitutes one of the potential anticorona viral strategies. Meanwhile, the trials are continuing, and it will be beneficial to introspect the past research on dexamethasone. In this article, we reviewed the pharmacogenomics of dexamethasone and the impact of dexamethasone on transcriptome and metabolome profiles. This compiled resource may be useful to decipher host genetic factors that might influence on the dexamethasone treatment.

## Dexamethasone: a synthetic corticosteroid

**Figure 1.**
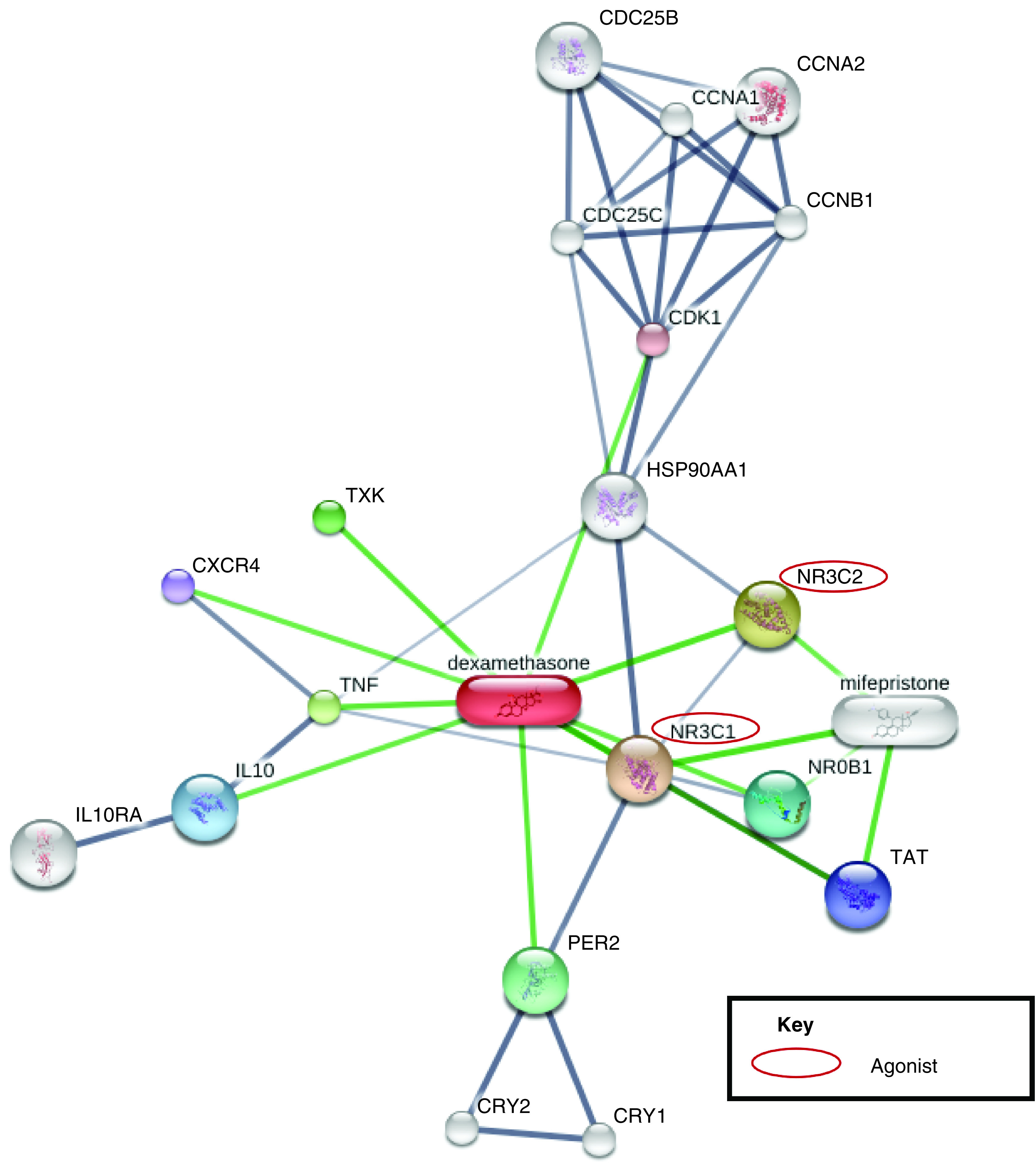
The interaction of dexamethasone with glucocorticoid receptors such as NR3C1 and NR3C2 and its secondary targets identified from STITCH database.

Dexamethasone is a potent anti-inflammatory drug that belongs to the category of synthetic adrenal corticosteroid [3]. Compared with other glucocorticoids, such as hydrocortisone and prednisone, dexamethasone is highly potent. It shows anti-inflammatory activity and is used to treat severe and acute inflammatory, allergic and immunological diseases. The drug binds to the nuclear steroid receptors and interferes with apoptotic pathways and NF-kB activation [12]. Dexamethasone, a glucocorticoid agonist, has the potential to penetrate the CNS [13]. The drug is also prescribed alone to manage cerebral edema and is used in combination with tobramycin for treating corticosteroid-responsive inflammatory ocular conditions [13]. The drug can cross cell membranes in unbound form and has an affinity for specific glucocorticoid receptors (GRs) such as NR3C1 and NR3C2 (Figure 1) [14]. The complex of dexamethasone and GR binds to glucocorticoid response elements and results in alteration of the transcription process [15]. This leads to interference in the inflammatory response, inhibition of leukocyte infiltration, edema reduction and humoral immune response suppression. Any alterations in the GRs or the glucocorticoid response element can affect dexamethasone binding on the receptor and produce varied effects.

The drug–gene interactions of the dexamethasone with the interacting receptor or enzymes have been shown in Figure 1. This was formulated using the STITCH database [14], where dexamethasone drug was used as the input drug and its interacting genes were extracted. According to the figure, the confidence score of dexamethasone interaction with NR3C1 and NR3C2 is high that suggests the drug has a binding affinity for these receptors.

Orally administered dexamethasone is absorbed by 61–86% [16]. The rate of absorption is slower via the intramuscular than the intravenous route [17]. The metabolism of dexamethasone takes place in the liver primarily by *CYP3A4* and to a lesser extent by *CYP3A5*. By the action of the CYP3A4 enzyme, dexamethasone is hydroxylated to 6α- and 6β-hydroxydexamethasone [18]. Corticosteroid 11-beta-dehydrogenase isozyme 2 enzyme converts dexamethasone to 11-dehydrodexamethasone, which can be reconverted by corticosteroid 11-beta-dehydrogenase isozyme 1. Thus, the genetic variations and the drugs that can modulate *CYP3A4* and *CYP3A5* gene function may affect the pharmacokinetics of dexamethasone. The half-life of dexamethasone in adults is 4 h when administered orally, whereas the half-life ranges from 1 to 5 h when administered intravenously [16]. The route of elimination is through urine.

Like other corticosteroids, dexamethasone also binds to GRs and inhibits pro-inflammatory signals [19], and duration of the effect action varies depending upon the route of administration [17]. The effect of dexamethasone includes a short-term decrease in permeability of capillaries and vasodilation and a decrease in migration of leukocytes to the inflammation site [19]. Dexamethasone also interferes with the demargination and apoptosis of neutrophils. Reports suggest that inflammatory transcription factors, such as NF-kB, are inhibited, and anti-inflammatory genes, such as IL-10, are activated by dexamethasone [20,21].

## Dexamethasone & GR polymorphisms

The impact of genetic variations in GRs on dexamethasone effects has been evaluated in few studies. Niu *et al.* (2009) studied the polymorphisms in *NR3C1* (GRα) gene and identified 108 polymorphisms [22]. Among these polymorphisms, the minor allele frequency of nine nonsynonymous single nucleotide polymorphisms (SNPs) and four synonymous SNPs was more than 5% [22]. Four common polymorphisms in *NR3C1* gene such as rs6190 (ER22/23EK), rs56149945 (N363S), rs41423247 (BclI) and rs6198 (9beta) have been linked to dexamethasone response [23–27]. The BclI G allele and 363S allele were associated with increased response [23–25], whereas the 22/23EK allele was related to decreased drug response [26]. A low dose of dexamethasone showed sex-specific association of adrenocorticotropic hormone and salivary cortisol suppression with 9beta AG genotype [27]. The rs5522 and rs2070951 in the *NR3C2* gene also show sex-specific modulation corticoids under dexamethasone treatment [28]. The rs5522 showed blunted suppression in males with homozygous AA genotype, whereas rs2070951 was associated with enhanced suppression in females and impaired suppression in male G-allele carriers [28]. Although these reports suggest genetic variations in GRs affect dexamethasone response, these in relation to COVID-19 affected individuals remain to be evaluated.

## Pharmacogenomics of dexamethasone

**Figure 2.**
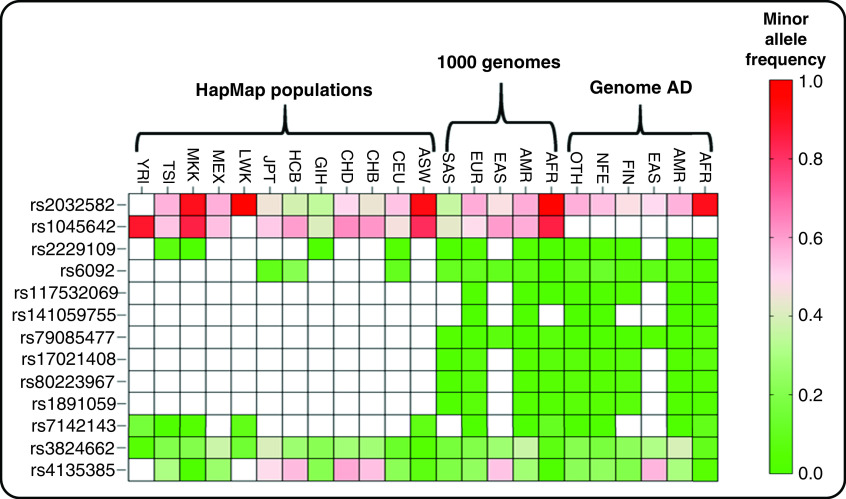
The distribution of minor allele frequency of clinically annotated variants for dexamethasone across various populations. 1000 Genome populations: AFR: African; AFR: African/African American; AMR: Ad Mixed American; AMR: Latino/Admixed American; ASW: African ancestry in Southwest United States; CEU: Utah residents with Northern and Western European ancestry from the CEPH collection; CHB: Han Chinese in Beijing, CHD: Chinese in Metropolitan Denver, Colorado; China; EAS: East Asian; EUR: European; FIN: Finnish; GIH: Gujarati Indians in Houston, Texas; JPT: Japanese in Tokyo, Japan; LWK: Luhya in Webuye, Kenya; MKK: Maasai in Kinyawa, Kenya; MXL: Mexican ancestry in Los Angeles, California; NFE: Non-Finnish European; OTH: Other (population not assigned). HapMap populations: SAS: South Asian. Genome Aggregation Database: TSI: Toscani in Italia; YRI: Yoruba in Ibadan, Nigeria.

The variation in response to dexamethasone has been reported in the literature. Thirteen SNPs have been clinically linked to dexamethasone (Table 1). The frequency of these variations varies across different populations (Figure 2). Among these variations, rs2032582 and rs1045642 in the *ABCB1* gene show the highest frequency of risk alleles in different populations of genome aggregation database [29], 1000 Genomes populations [30] and HapMap populations [31].

**Table 1.** List of clinically annotated variants for dexamethasone.

S. N.	Gene	SNP ID	Location	Consequence	Nucleotide change	Effect	Disease
1	*ABCB1*	rs2032582	chr7:87531302	Missense variant	A/C	Efficacy	Multiple myeloma
		rs1045642	chr7:87509329	Missense variant	A/G	Efficacy	Multiple myeloma
		rs2229109	chr7:87550493	Missense variant	C/T	Efficacy	Multiple myeloma progression-free survival
2	*SERPINE1*	rs6092	chr7:101128436	Missense variant	G/A	Toxicity/ADR	Precursor cell lymphoblastic leukemia-lymphoma
3	*DOK5*	rs117532069	chr20:54684529	Regulatory region variant	G/A	Toxicity/ADR	Osteonecrosis, precursor cell lymphoblastic leukemia-lymphoma
4	*LINC00251*	rs141059755	chr8:65195370	Intergenic variant	A/G	Toxicity/ADR	Osteonecrosis, precursor cell lymphoblastic leukemia-lymphoma
5	*BMP7*	rs79085477	chr20:57126159	Intron variant	C/T	Toxicity/ADR	Osteonecrosis, precursor cell lymphoblastic leukemia-lymphoma
6	*PROX1-AS1*	rs17021408	chr1:213769895	Intron variant	T/C	Toxicity/ADR	Osteonecrosis, precursor cell lymphoblastic leukemia-lymphoma
		rs80223967	chr1:213770336	Intron variant	A/G	Toxicity/ADR	Osteonecrosis, precursor cell lymphoblastic leukemia-lymphoma
		rs1891059	chr1:213772666	Intron variant	G/A	Toxicity/ADR	Osteonecrosis, precursor cell lymphoblastic leukemia-lymphoma
7	*PYGL*	rs7142143	chr14:50936813	Intron variant	T/C	Efficacy	Precursor cell lymphoblastic leukemia-lymphoma
8	*GATA3*	rs3824662	chr10:8062245	Intron variant	C/A	Efficacy	_
9	*CTNNB1*	rs4135385	chr3:41237949	Intron variant	A/G	Efficacy/toxicity/ADR	Multiple myeloma

ADR: Adverse drug reactions; SNP: Single nucleotide polymorphism; S.N.: Serial number.

Adapted with permission from [32].

Early studies speculated that dexamethasone treatment might influence the expression of multidrug resistance transporters [33]. An investigation on the rat blood–brain barrier under the influence of dexamethasone treatment highlighted increased expression of *ABCG2*, *PGP* and *ABCC2* genes. The results suggested that the use of corticosteroids, such as dexamethasone as adjuvants, may increase MDR transporter expression in the treatment of brain tumors [33]. Mancea *et al.* (2012) showed that dexamethasone could itself induce a fourfold increase in expression of the *ABCB1* gene in cytotrophoblast cells [34]. Another study showed that dexamethasone induces *ABCB1* gene expression in CCRF-CEM cells, a human T-lymphoblastoid cell line [35]. *ABCB1* gene belongs to ATP Binding Cassette Subfamily B Member 1 and is involved in the translocation of phospholipids and drugs across different membranes [36–38]. The gene is overexpressed in cancer cell lines and acute myeloid leukemia. SNPs in the *ABCB1* gene, such as rs2032582, rs1045642 and rs2229109, have been associated with altered dexamethasone response [39]. The individuals with CC genotype for rs2032582 showed decreased survival in compared with AC or AA genotype individuals [39]. However, the rs1045642 AA-genotype individuals showed better survival on treatment with dexamethasone [39]. It was recently shown that individuals with rs2229109 CC-genotype have shorter progression-free survival when treated with dexamethasone and lenalidomide [40].

*SERPINE1* gene codes for a serine protease inhibitor primarily inhibit the urokinase-type plasminogen activator and tissue-type plasminogen activator. The protein is required to downregulate fibrinolysis to control blood clot degradation [41–43]. It regulates cell migration and stimulates keratinocyte migration during injury repair [44]. The risk of osteonecrosis in pediatric individuals with acute lymphoblastic leukemia (ALL) and AA-genotype for rs6092 is more when treated with dexamethasone in comparison with GG-genotype [45].

Karol *et al.* (2016) studied the genetic factors associated with osteonecrosis in children undergoing treatment for ALL [46]. This study identified rs117532069 in *DOK5*, rs141059755 *LINC00251*, rs79085477 in *BMP7* and rs17021408, rs80223967 and rs1891059 in *PROX1-AS1* in association with osteonecrosis [46]. The pediatric individuals with AA genotype for rs117532069 *DOK5* showed an increased probability of developing osteonecrosis when they were undergoing treatment with anticancer drugs and dexamethasone in comparison with AG or GG genotypes [46]. *DOK5*, plays an important role in MAP kinase pathway activation. The *DOK5* gene is also known to mediate neurite outgrowth through *RET*, which is a proto-oncogene. Karol *et al.* (2016) were also able to successfully replicate and validate rs141059755 in *LINC00251* and rs79085477 in the *BMP7* gene [46]. *BMP7* gene codes for the bone morphogenetic protein 7, which is vital for bone and cartilage formation. The gene is also responsible for bone homeostasis and calcium regulation. Dexamethasone can induce the proliferation of muscle cells and bone-marrow-derived cells [47]. Along with dexamethasone treatment, the *BMP7* expression showed a synergistic effect on osteogenic differentiation of human embryonic stem cell (hESC)-derived mesenchymal stem cells [48]. *PROX1-AS1* codes for antisense RNA of *the PROX1* gene is a transcriptional factor important for determining cell fate in many organs [49].

The risk of precursor cell lymphoblastic leukemia relapse was also accessed in children undergoing dexamethasone or methotrexate, asparaginase treatment [50]. It was observed that children with CC and CT-genotypes for rs7142143 *PYGL* showed an increased risk of relapse in comparison with TT-genotype [50]. In the same study, it was shown that two SNPs in the *ABCB1* gene, namely, rs10264856 and rs4728709, correlated with a higher clearance of dexamethasone and increased risk of relapse [50]. The relapse risk was also associated with rs3824662 risk allele A in the *GATA3* gene and early treatment response in Ph-like ALL [51]. In the case of multiple myeloma, the AA-genotype of the *CTNNB1* gene (that encodes for catenin beta 1) correlated with an increased response to dexamethasone, cyclophosphamide, and thalidomide treatment in comparison with GG and AG genotypes [52].

## Dexamethasone-induced transcriptome changes

During the development of organs, such as the thyroid, kidneys, lungs, gut, brain and pituitary, there is a surge of glucocorticoids [53,54]. Duma *et al.* (2010) have shown the sex-specific effects of glucocorticoids in the liver [55]. Frahm *et al.* (2016) studied the effects of dexamethasone on the hypothalamus cells from male and female C57BL/6 mice [56]. They identified glucocorticoids’ known targets, such as *Fkbp5*, *Cftr*, and *Fam107a* [56]. They further found that the expression of genes such as *Hif3α* was robustly altered by dexamethasone treatment in both male and female cells [56]. Sex-specific changes in gene expression were also observed [56]. A total of 137 genes, including *Map7, lincRNA H19* and *miR675* genes, were differentially expressed in female cells, whereas 53 genes, including *Klf4*, were differentially expressed in male cells [56]. Corticosteroids are potent anti-inflammatory agents and are known to regulate the transcription of cytokine genes [57]. Dexamethasone inhibits IL-6 gene expression at lower concentrations and IL-6 receptor expression at higher concentrations [58]. The effects of dexamethasone on human multiple myeloma cells were reversed with IL-6 treatment [58]. Besides IL-6, IL-2 levels were also modulated dexamethasone [59]. Dexamethasone strongly inhibits the proliferation of effector T cells and weakly inhibits the proliferation of regulatory T cells [59]. The inhibitory effects of dexamethasone on T cells are under the modulation of IL-2 and can be restored [59]. Recently, the impact of dexamethasone was tested in combination with a gain of function mutation in GR Ala610Val [60]. About 30% of the genes were affected due to dexamethasone treatment. The study showed that even in the absence of immune stimulus, the drug showed a maximum impact on the expression of genes related to inflammation [60]. In combination with the genotype, dexamethasone affected protocadherins, particularly *PCDHB7*. The administration of dexamethasone at lower doses induced better glucose response in carriers of Val receptors [60]. This finding also suggested that variations in GR genes modulate the response to glucocorticoids such as dexamethasone.

## Dexamethasone-induced metabolite changes

Studies conducted on metabolites in an individual’s body to assess various drugs’ metabolism and understand their pharmacokinetics are referred to as pharmacometabolomic studies [61]. Bordag *et al.* (2015) studied and presented the changes observed in the metabolite profile (150 metabolites) of healthy male volunteers under the influence of dexamethasone [62]. They observed several metabolites that were significantly altered at different times after the drug administration [62]. The observed changes in metabolic profile were proposed to be linked to systemic side effects. Although during the study, no clinical side effects were observed [62]. However, the study observed deviations in suppression of steroid production, catecholamine alteration, polyunsaturated fatty acids levels and trans-4-hydroxyproline, which may be related to adrenal gland depression, psychological problems, infection, atherosclerosis, hypertension, diabetes and osteoporosis [62]. Malkawi *et al.* (2018) evaluated the changes in the metabolite profile of an established animal model with known dexamethasone-related side effects [63]. The significant variations reported were in the levels of alanine, hydroxyproline, tryptophan, kynurenine, phenylalanine and tyrosine metabolites in dexamethasone-treated rats [63]. These disturbances were correlated with a reduction in weight gain, dyslipidemia, hyperglycemia and abnormal bone turnover [63].

## Repurposing of dexamethasone as a potential drug against COVID-19

The immunomodulatory drugs are being used in addition to antiviral therapy for COVID-19 [64]. This is mainly to reduce death and suppress hyperinflammation [64]. There is a history of corticosteroids as immunomodulatory drugs in the severe forms of coronavirus disease, such as MERS, SARS and COVID-19 [65–67]. The demand for dexamethasone has surged after the RECOVERY trial showed a successful reduction of deaths in critical COVID-19 patients [68]. In the case of patients suffering from a less severe form of COVID-19, no positive outcomes were observed. A randomized controlled trial (RCT) showed a reduction in mortality of acute respiratory distress syndrome (ARDS) patients [69]. The dexamethasone usage for COVID-19 treatment has been supported by recent observations, such as preventing disease severity with short-term course of the drug leading to a further reduction in ICU stay of patients [70]. The dexamethasone drug is useful in suppressing the hyperactive immune response that aids in overcoming serious manifestation in COVID-19 patients. However, due to the lack of adequate RCTs, the use of corticosteroids is still controversial. At present, WHO has given a strong recommendation for dexamethasone in severe and critical COVID-19 patients but not in nonsevere COVID-19 patients [71]. However, due to the lack of adequate RCTs, the use of corticosteroids is still controversial. For the proper application of the drug, factors such as inter-individual variations and the impact of dexamethasone on the individual’s transcriptome and metabolome must be carefully examined and weighed.

## Conclusion

The dexamethasone drug has been in use for a long time. The WHO has recommended the prescription of 6 mg of dexamethasone dose for severe and critical COVID-19 patients for 7–10 days. Despite the promising preliminary results of dexamethasone use in chronic COVID-19 patients, individuals’ effects must be carefully weighed. This must include the overall transcriptome and metabolome effects that can impact the physiology of COVID-19 patients. The known pharmacogenomic variants may also have an impact on dexamethasone therapy.

## Future perspective

The urgent need to develop a cure for COVID-19 has pushed barriers to regulatory controls of RCTs. Meanwhile, the use of immunomodulatory drugs such as dexamethasone, which has shown to reduce mortality in severe COVID-19 patients, is being encouraged. However, selecting the COVID-19 patients for treatment with dexamethasone based on the severity of the disease alone may still not be appropriate. Considering the pharmacogenomic knowledge of the dexamethasone and the pharmacodynamic nature of the drug may further aid in decreasing the mortality rate in severe COVID-19 patients in the future. This will help in delivering precision medicine to severe COVID-19 cases.

**Executive summary**

**Background**

- There is a history of the use of corticosteroids as immunomodulatory drugs in the severe forms of coronavirus disease, such as MERS, SARS and COVID-19.

**Pharmacogenomics of dexamethasone: a synthetic corticosteroid**

- The dexamethasone usage for COVID-19 treatment has shown encouraging preliminary results in severe patients.
- The inter-individual variations in response to dexamethasone must be considered before it applies for COVID-19 treatment.

**Dexamethasone-induced transcriptome changes**

- The variation in response to dexamethasone has been reported previously and linked to 13 single nucleotide polymorphisms.
- Dexamethasone induces significant changes in the transcriptome of individuals undergoing therapy.

**Dexamethasone-induced metabolite changes**

- Besides, dexamethasone also causes changes in metabolite levels.

**Repurposing of dexamethasone as a potential drug against COVID-19**

- Consideration of pharmacogenomic aspects will derive better therapy outcomes in COVID-19 patients.
